# Oil body-associated hazelnut allergens including oleosins are underrepresented in diagnostic extracts but associated with severe symptoms

**DOI:** 10.1186/2045-7022-4-4

**Published:** 2014-02-02

**Authors:** Laurian Zuidmeer-Jongejan, Montserrat Fernández-Rivas, Marcel GT Winter, Jaap H Akkerdaas, Colin Summers, Ans Lebens, André C Knulst, Piet Schilte, Peter Briza, Gabriele Gadermaier, Ronald van Ree

**Affiliations:** 1 Department of Experimental Immunology, Academic Medical Center, Meibergdreef 9, Amsterdam 1105 AZ, The Netherlands; 2 Allergy Department, Hospital Clínico San Carlos, IdISSC, Madrid, Spain; 3 CM&MC, Manchester, United Kingdom; 4 Department Dermatology and Allergology, University Medical Centre Utrecht, Utrecht, The Netherlands; 5 Department of Pediatrics, Medical Center Alkmaar, Alkmaar, TheNetherlands; 6 Christian Doppler Laboratory for Allergy Diagnosis and Therapy, Department of Molecular Biology, University of Salzburg, Salzburg, Austria; 7 Department of Otorhinolaryngology, Academic Medical Center, Amsterdam, The Netherlands

**Keywords:** Food allergy, IgE-mediated, Oil bodies, Oleosins, Hazelnut

## Abstract

**Background:**

Oil body-associated allergens such as oleosins have been reported for important allergenic foods such as peanut, sesame and hazelnut. Here we investigate whether oil body associated proteins (OAPs) are linked with specific clinical phenotypes and whether they are represented in skin prick test (SPT) reagents.

**Methods:**

A hazelnut OAP fraction was characterized by mass-spectrometry (MS) to identify its major constituents. Polyclonal rabbit antibodies were generated against hazelnut OAPs. The presence of OAPs in commercially available hazelnut SPTs was studied by immunoblot and spiking experiments. OAP-specific IgE antibodies were measured in sera from patients with a convincing history of hazelnut allergy by RAST (n = 91), immunoblot (n = 22) and basophil histamine release (BHR; n = 14).

**Results:**

Hazelnut OAPs were analysed by MS and found to be dominated by oleosins at ~14 and ~17 kDa, and a 27 kDa band containing oleosin dimers and unidentified protein. In 36/91 sera specific IgE against hazelnut OAPs was detected, and confirmed to be biologically active by BHR (n = 14). The majority (21/22) recognized the oleosin bands at 17 kDa on immunoblot, of which 11 exclusively. These OAP-specific IgE responses dominated by oleosin were associated with systemic reactions to hazelnut (OR 4.24; p = 0.015) and negative SPT (*χ*^2^ 6.3, p = 0.012). Immunoblot analysis using OAP-specific rabbit antiserum demonstrated that commercial SPT reagents are virtually devoid of OAPs, sometimes (3/9) resulting in false-negative SPT. Spiking of SPT reagents with OAP restored serum IgE binding of these false-negative patients on immunoblot at mainly 17 kDa.

**Conclusion:**

Hazelnut allergens found in oil bodies dominated by oleosin are associated with more severe systemic reactions and negative SPT. Defatted diagnostic extracts are virtually devoid of these allergens, resulting in poor sensitivity for detection of IgE antibodies against these clinically relevant molecules.

## Background

Food allergic reactions after hazelnut ingestion are frequently observed [1,2]. In Central and Northern Europe, allergy to hazelnut is mostly associated with birch-pollen allergy and is almost exclusively mild and restricted to the oral cavity [3]. Primary sensitization to the major birch pollen allergen Bet v 1 results in cross-reactivity to its homologue in hazelnut, Cor a 1[4,5]. Non-pollen related hazelnut allergy more frequently induces severe systemic reactions which can already be observed in young children that have not (yet) developed inhalant allergies [6,7]. These severe reactions are the dominant clinical presentation in areas without significant exposure to birch pollen like Spain[8]. Non-pollen related allergens that have been identified in hazelnut so far are the non-specific lipid transfer protein (LTP), Cor a 8 [9] and homologues of the major peanut allergens Ara h 1 (7S vicilin) Ara h 2 (2S albumin) and Ara h 3 (11S globulin), the hazelnut storage proteins Cor a 11, Cor a 14 and Cor a 9 respectively [10-12]. The latter two have recently been demonstrated to be associated with more severe objective symptoms in hazelnut allergy[11,13].

Although sufficient quantities of these water-soluble allergenic storage proteins are present in diagnostic reagents [14], a substantial number of (tree)nut and seed-allergic patients is not diagnosed adequately using commercial SPT and in vitro reagents ([15,16]; pers. communication G. Lack). For Cor a 8, it has been reported that this allergen is virtually absent in some commercial SPT reagents [17]. The low levels of LTP can be explained by the extraction protocols used for peanut or hazelnut, which are commonly carried out at neutral pH where LTPs are optimally soluble at acidic pH [18,19]. Another characteristic of extraction procedures used for legumes, seeds and nuts is that de-fatting steps are included. These types of foods however contain oil bodies. A major protein in oil bodies is oleosin, a protein constituting around 10-20% of the total seed protein [20-22]. Oleosins are alkaline proteins (pI ± 10) with molecular weights from 15 to 24 kDa [23], which stabilize triacylglycerol containing oil bodies in the cytoplasm [22]. Oil bodies are an energy source for growth and germination of seedlings. Oleosins are embedded in the oil body with a highly conserved hydrophobic central domain [24], whereas the hydrophilic alpha-helical N-terminus and amphipathic alpha-helical C-terminus, which is conserved among many oleosins [20-22], are in contact with the aqueous cytoplasm[25].Oleosins have been identified as allergens in peanut, sesame seed and hazelnut [16,26,27].

We hypothesize that patients with convincing (sometimes severe) hazelnut allergy but negative diagnostic tests, may often have specific IgE antibodies against oil body-associated proteins (OAPs) such as oleosins, allergens that are largely removed from diagnostic food extracts during de-fatting steps. The aim of this study was to test commercially available skin prick test (SPT) reagents with respect to the presence of OAPs, and to use purified OAPs from hazelnut to evaluate the clinical importance of OAP-specific IgE antibodies using sera from well-characterized patients with a convincing history and/or positive DBPCFC for hazelnut. Immunoblot analysis in conjunction with mass spectrometry was used to establish the role of oleosins as the major allergenic components of OAPs.

## Material and methods

### Patients

**Figure 1:**
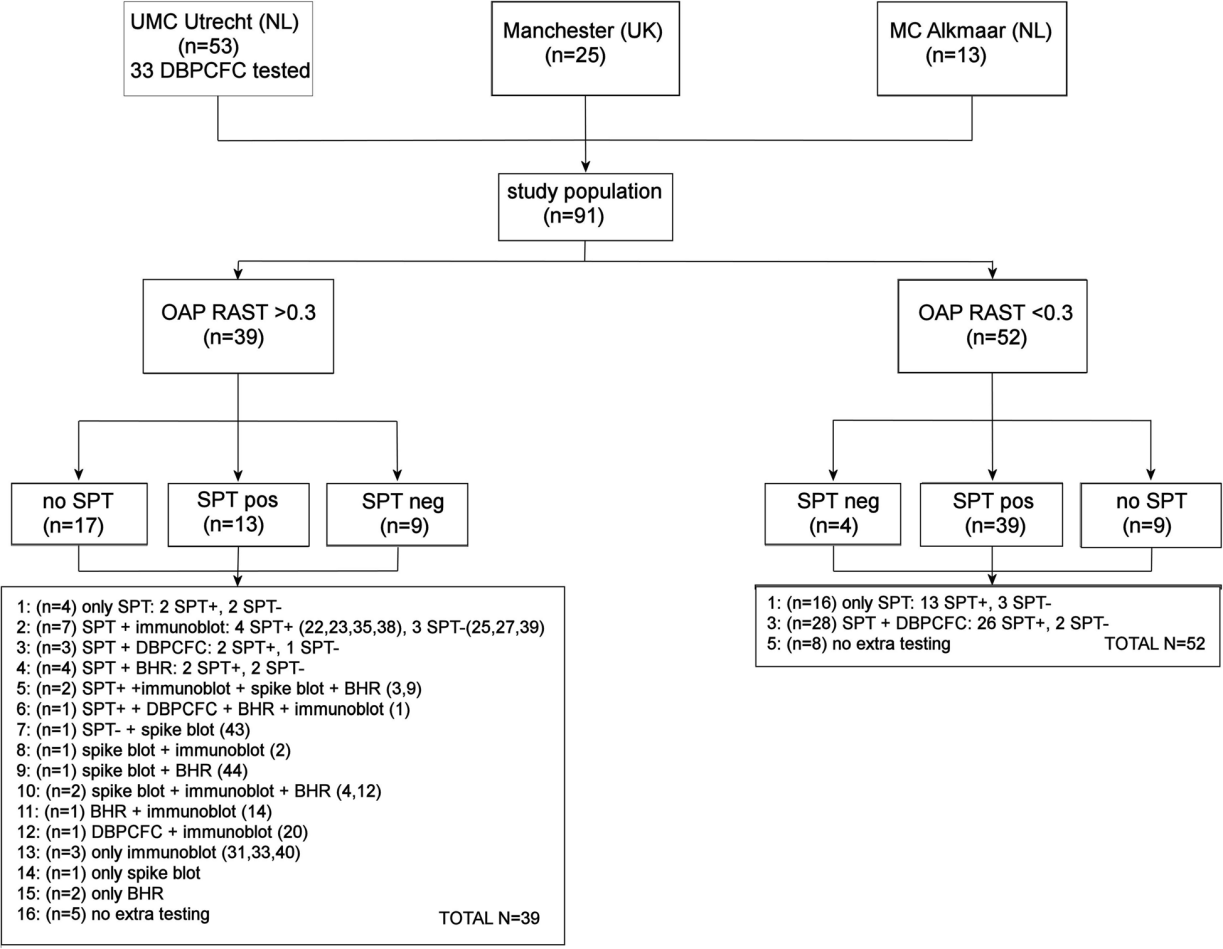
**Flowchart indicating the source of the sera and which experiments they were used.** If sera were used for blotting experiments, the serum numbers are indicated.

We used a retrospectively collected panel of 91 sera of patients with a convincing history of hazelnut ingestion-related symptoms that were recruited via the outpatient clinics of the University Medical Centre Utrecht (n = 53) and the Medical Centre Alkmaar (n = 13, The Netherlands) and the Manchester Royal Infirmary (n = 25, UK). All patients gave written informed consent for using their serum for research purposes. The patients from Utrecht were all collected in the framework of a study on hazelnut allergy, and in 30/53 this was confirmed by DBPCFC [28]. The patients from Alkmaar were collected in the framework of a collaboration between Sanquin (Amsterdam) and the Medical Centre Alkmaar on severe tree nut allergies, in the period from 1980–2005. The medical history and diagnostic tests were carefully recorded by the physician and retrieved retrospectively for this study. The patients from the UK were part of the serum collection of C. Summers and R. Pumphrey, who also had detailed histories recorded. A summary of the different diagnostics performed with these sera is outlined in Figure 1.

Severity of recorded allergic reactions was classified into five categories, essentially following the Mueller procedure for venom allergies [29] and adapted amongst others by M. Fernandez-Rivas for food allergy. Grade 0 included local oropharyngeal reactions, known as oral allergy syndrome (OAS). Grades 1 to 4 were applied to systemic reactions: grade 1, skin involvement without angioedema; grade 2, skin involvement with angioedema and/or gastrointestinal symptoms; grade 3, any of the previous with respiratory symptoms; grade 4, any of the previous with cardiovascular symptoms. For dichotomous statistical analyses, grade 1–4 were taken together in one category systemic, opposed by grade 0 being local. Information on the severity of the reaction to hazelnut was available for 78/91 patients.

SPT were carried out following EAACI guidelines [30]. An SPT was considered positive if the mean wheal diameter was 3 mm (over the negative control) or the ratio to histamine wheal was ≥0.5. Hazelnut SPT data were available for 65/91 patients.

Ethical approval for the study was available in each of the participating centres.

### Hazelnut extracts

Unroasted hazelnuts were bought at a local food store. Non-defatted hazelnut extract was prepared as described previously [17]. Defatted hazelnut extract was made by shaking ground hazelnuts (3 times) with 30% (v/v) freon 141b NP (Promosol, Brussels, Belgium) for 1 hour and centrifugation for 20 minutes at 11,000 *g* before extraction like above. The commercially available SPT extracts used have been characterized and described previously [17] and were purchased from nine manufacturers: (A) ALK-Abello’ (Nieuwegein, The Netherlands), (B) Allergopharma (Reinbeck, Germany), (C) Allergy Laboratories of Ohio (Columbus, Ohio, USA), (D) Stallergènes, (Antony, France), (E) Artu Biologicals Europe, (Lelystad, The Netherlands), (F) Bayer (Elkhart, Ind., USA), (G) Greer Laboratories, (Lenoir, N.C., USA), (H) HAL Allergenen Lab. (Haarlem, The Netherlands) and (I) Nelco Laboratories (New York, N.Y., USA). All reagents were purchased as ready-for-use products and were diluted to a concentration of 250 μg/ml for immunoblots. For spiking experiments, extract D was used with or without approximately 5 μg OAPs.

### Oil body isolation

An oil body-enriched fraction was purified from hazelnut combining several methods described before [26,31]. Unroasted hazelnuts were bought at a local food store and ground 1:2 w/v in grinding buffer (GB; 1 mM EDTA, 10 mM KCl, 1 mM MgCl_2_, 2 mM DTT, 0.15 M tricine, 0.6 M sucrose, pH 7.5 with KOH) using a Waring blender (Waring commercial, Hartford, CT) for approximately 5′. The extract was filtered using rough-mazed gauze (HG Kompressen, Klinion) and layered with 1:1 v/v flotation medium (FB; GB with 0.4 M sucrose). After centrifugation (30′ at 10.000 × *g*) the fat pad was resuspended in detergent washing solution (GB but with 0.2 M sucrose, 0.1% Tween-20, 75 mM tricine) and layered with 1:1 v/v 150 mM Tricine (pH 7.5). After centrifugation, the fat pad was re-suspended in re-suspension buffer (RB; GB containing 2 M NaCl), layered with 1:1 v/v floating medium (RB containing 0.25 M sucrose) and centrifuged. The resulting fat pad was re-suspended in 9 M urea, mixed at RT for 10′, layered with 1:1 v/v 150 mM tricine (pH 7.5) and centrifuged as previously. The fat pad was re-suspended in GB, layered with 1:1 v/v FB and centrifuged. Finally, the fat pads were re-suspended in GB to a concentration of 100 mg lipid/ml.

### Defatting of oil-bodies

In order to purify oil body-associated proteins from the oil body enriched fraction, the oil bodies were defatted according to the method previously described [32]. After the last separation, the interphase was dried o/n in a fumehood before dissolving in PBS (precipitate remains). This fraction will be referred to as OAP (oil body-associated protein) fraction.

### Production of antibodies

For polyclonal antibody production, New Zealand White rabbits were immunized with ~50 μg purified hazelnut OAP fraction in Montanide ISA-50 (Seppic, Paris, France). The oil body fraction was filtered (Schleicher and Schuell, New Hampshire, USA) then both fractions were dialyzed to PBS and mixed 1:1 with 6.25% glycerol. Boosters were given at 4 week intervals. Plasma was collected at day 7, 9 and 11 (after boosters 4, 5 and 6), re-calcified and antibody titers were measured by RAST with Sepharose-coupled natural hazelnut OAPs. Rabbits’ sera were pooled and stored frozen at -20°C.

### Expression and purification of recombinant oleosin

Hazelnut-derived cDNA was used in a PCR with primers based on the published sequences of both hazelnut oleosin isoforms (primary accession numbers Q84T91 and Q84T21 [27]). After ligation of the fragments into the pET19b expression vector (Novagen, Merck, kGaA, Darmstadt, Germany) the sequence of the clones was confirmed (BaseClear Lab services, Leiden, The Netherlands). Both recombinant hazelnut oleosins (AY224679 and AY224599, Cor a 12 and Cor a 13 respectively) were expressed in *E.coli BL21* (DE3), in MagicMedia™ (Invitrogen, Carlsbad, CA, US). Since they were found to be predominantly in inclusion bodies, both isoforms were denatured by addition of 6 M guanidin-HCl and purified via Ni^+^- chelate affinity chromatography using a HIS-trap HP 1 ml column (GE Healthcare, Little Chalfont, Buckinghamshire, UK). Elution was performed with a linear gradient from 20–500 mM imidazole. The oleosins eluted in one main peak at a concentration of 300 mM imidazole and were first precipitated with ethanol [33] to remove the imidazole and then dissolved in water. As we did not manage to isolate significant amounts of Cor a 13, only Cor a 12 was used for the inhibition experiment.

### SDS-PAGE/immunoblotting

SDS-PAGE and immunoblotting were performed as described before [11] with 100 μL human serum and horseradish peroxidise (HRP)-labelled goat anti-human IgE (KPL, Gaithersburg, MD, USA) as secondary antibody. Rabbit anti-OAPs was diluted 1:11.000 in PBS/10 mM EDTA/0.3% BSA/0.1% Tween-20, and detection was done with radio-labelled sheep antibodies against rabbit IgG, as described previously [34]. For blot inhibition studies, 100 μL of the inhibitor (10 μg/ml) was added together with the patient’s serum.

### Basophil histamine release assay (BHR)

BHR was carried out using the so-called stripped basophil protocol as described earlier [35,36]. In short, white blood cells were isolated from blood of a non-allergic donor by Percoll centrifugation and stripped from IgE by lactic acid treatment. Subsequently cells were re-sensitized with patient’s serum. Histamine release was performed with a dilution series of the OAP fraction. Histamine was measured by the fluorometric method essentially as described by Siraganian [37]. Stripped cells were used as a negative control. As positive control anti-IgE was used. The protocol was approved by the medical ethical committee (MEC) of the Amsterdam Medical Centre under project number: MEC97/030.

### RAST

For application in RAST, 3 ml of the water-soluble part of the OAP fraction (approximately 25 μg/ml as determined using the Pierce BCA protein assay (Pierce, Rockford, IL) was coupled to 100 mg CNBr-activated Sepharose 4B (Amersham-Pharmacia-Biotech, Uppsala, Sweden). RAST was performed as described previously [38].

### ImmunoCAP

CAP analysis was performed using the UniCAP®100 according to the manufacturer’s instructions (Thermo Fisher Scientific, Uppsala, Sweden).

### ESI-QTOF mass spectrometry

Identification of protein bands was performed by LC-MS/MS after separation of OAP fraction by SDS-PAGE. Bands were excised from CBB R-250 stained 15% polyacrylamide gels and in-gel digested using the ProteoExtract All-In-One Trypsin Digestion Kit (Calbiochem, San Diego, USA). Resulting peptides were separated by reversed phase capillary HPLC (Nanoease Symmetry 300 trap column and Nanoease Atlantis dC18 separating column, connected via a ten-port stream select valve; Micromass-Waters, Milford, USA). The flow rate was adjusted to 300 nL/min by T-splitting. Peptides were eluted with an ACN gradient (solvent A: 0.1% v/v formic acid/5% v/v ACN, solvent B: 0.1% v/v formic acid/95% v/v acetonitril; 5–45% B in 90 min) and directly infused into a Global Ultima Q-Tof instrument with electrospray ionization (Micromass QT of Global Ultima mass spectrometer, Waters, Milford, USA). For sequence analysis, the instrument was calibrated with the fragment ions of [Glu]-Fibrinopeptide B (Sigma, Steinheim, Germany). Data were acquired in the Data Directed Analysis (DDA) mode. Survey and fragment spectra were analyzed using the software PLGS version 2.2.5 (Waters) with automatic and manual data verification. For sequence identification, a combined Swiss-Prot/TrEMBL database was used.

### Statistical analysis of clinical data

Qualitative variables (gender, atopic dermatitis and respiratory allergies) are presented as frequency (percent). For quantitative variables such as hazelnut CAP and oleosin RAST median and interquartile range (IQR) are given. Nonparametric correlations (Spearman’s rho) between severity grade and hazelnut CAP and oleosin RAST were calculated. For univariate and multivariate analysis clinical severity was dichotomised as local (grade 0) or systemic (grades 1 + 2 + 3 + 4). In the univariate analysis the association between severity and the clinical variables, hazelnut CAP and OAP RAST was analysed by chi-square test. Associations with p-values below 0.10 from the univariate analysis were further analyzed in a logistic regression model to predict systemic reactions. Association between SPT (positive/negative) and the OAP RAST (positive/negative) was analysed by chi-square test. Calculations were performed using SPSS (version 15, SPSS Inc., 2001, Chicago, USA). P-values <0.05 were considered significant.

## Results

### Purification of OAP from hazelnut

**Figure 2:**
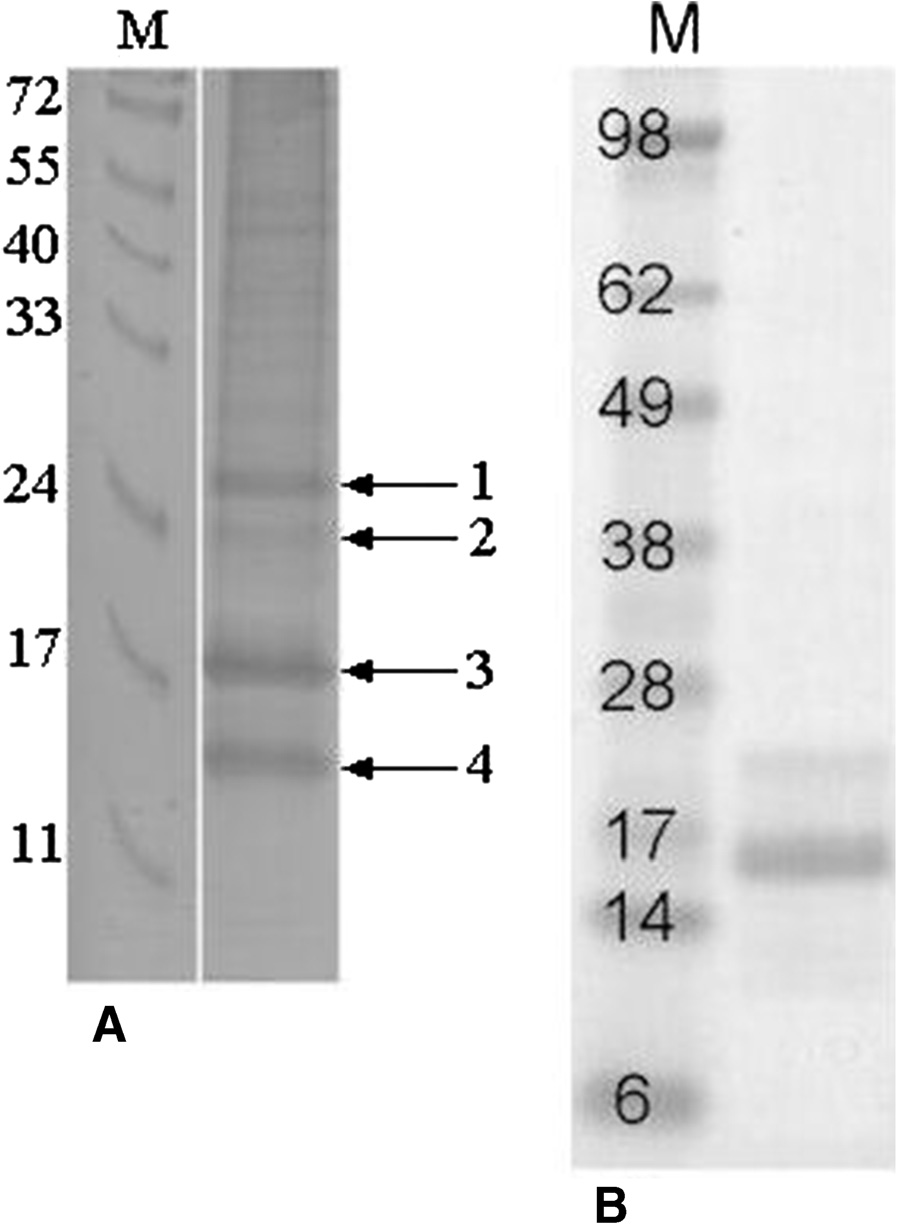
**Coomassie stained gels. A**; natural hazelnut OAP fraction. Indicated are the bands identified by mass-spectrometry. (1) contained little amounts of both oleosins, but the main constituent remained unidentified, (2) 11S globulin (AAL73404, Cor a 9) and (3) and (4) mainly consisted of both oleosins, AY224679 (Cor a 12) being the most abundant. In addition, a protein with similarity to caleosins was detected in these bands. **B**; Purified recombinant oleosin Cor a 12. Marker sizes are indicated.

The OAP fraction of hazelnut was analyzed by SDS-PAGE (Figure 2A). Three major protein bands were detected around 14, 17 and 27 kDa, respectively. In addition, there was a minor band at ~24 kDa and unclear bands of higher molecular weight. LC/MS-MS analysis identified the 14 and 17 kDa bands to be predominantly oleosins (Cor a 12 and Cor a 13) with traces of caleosin. The main constituent of the 27 kDa band could not be identified, but it also contained traces of both oleosins, presumably migrating as dimers. The faint 24 kDa band was found to contain an 11S globulin subunit (AAL73404, Cor a 9).

### IgE reactivity to the OAP fraction by RAST and immunoblot

**Figure 3:**
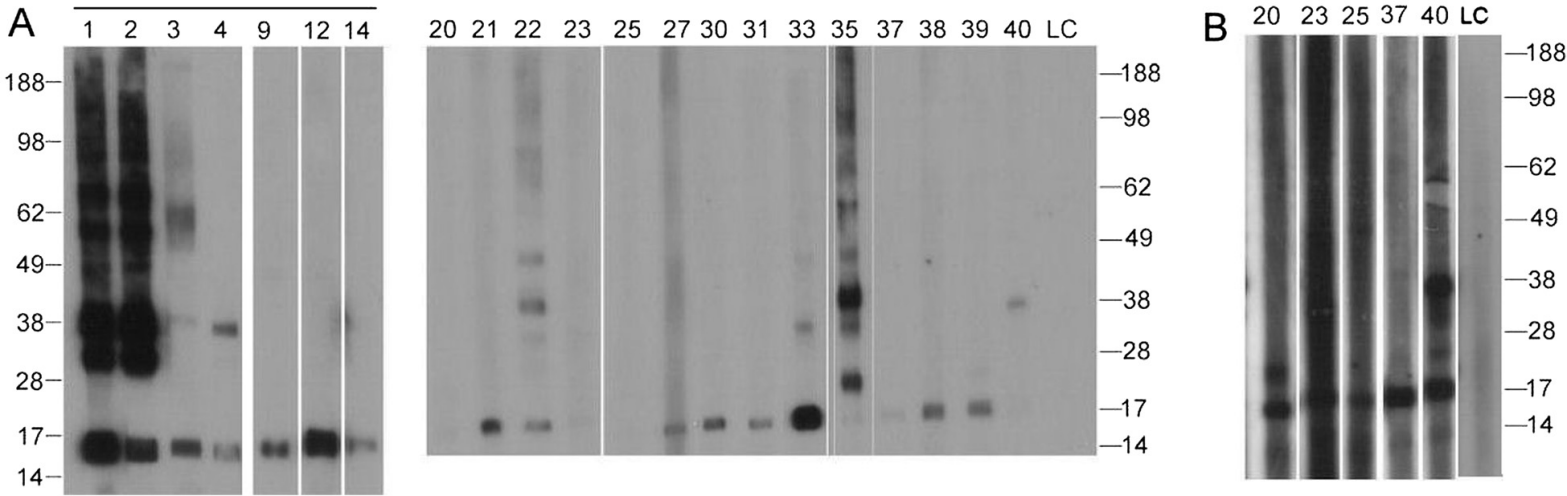
**Immunoblot showing reactivity of hazelnut allergic patients’ sera to the natural hazelnut OAP fraction. A**; 5′ exposure, **B**; 3 days exposure of selected sera. Sera tested in histamine release are indicated with a horizontal line above the serum nr. LC, label control. Marker sizes in kDa are indicated on the sides.

A panel of 91 sera was tested for IgE-reactivity to the OAP fraction in RAST. 36/91 had sIgE > 0.35 kU/L with a mean IgE-reactivity of 6.3 kU/L (ranging from 0.35-35.1 kU/L). To establish which constituents of the OAP fraction were recognized by IgE, all available sera with a sIgE > 1 kU/L (n = 21) were also analysed by immunoblot with the OAP fraction. In the OAP fraction, 15 sera clearly recognized the 17 kDa oleosin band (Figure 3A). At longer exposure, an additional 5 sera recognized the oleosin band (Figure 3B). Around 11/21 were selectively reactive with the 17 kDa band (see Figure 2A).

**Figure 4:**
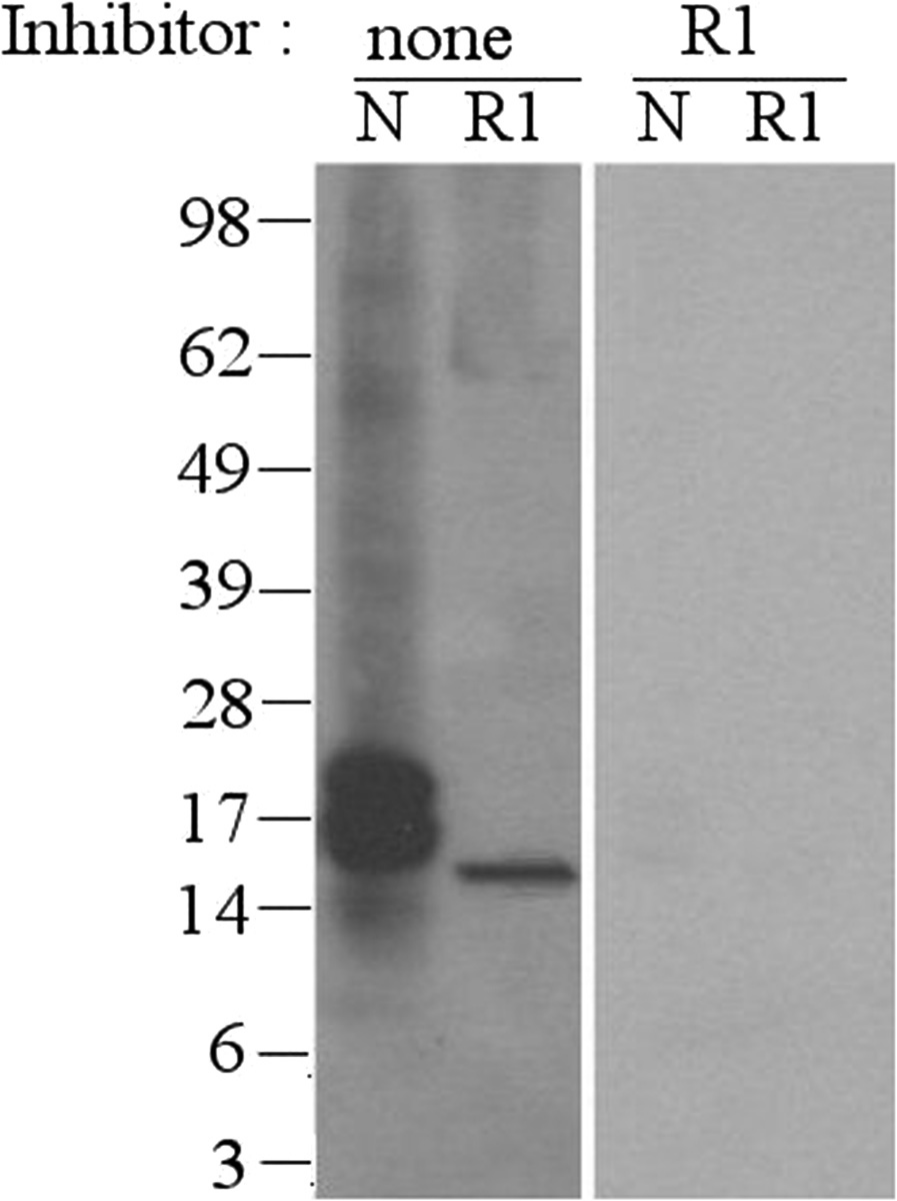
**Immunoblot-inhibition: Two identical immunoblots loaded with (N): nOAP fraction and (R1): rCor a 12.** The nature of the inhibitor is indicated above. Detection with a hazelnut-allergic patient’s serum (serum nr. 9, also in Figures 2 and 6). The marker sizes are indicated, the exposure time was identical.

Hazelnut oleosin Cor a 12 (AY224679) was expressed in E.coli and purified (up to ~80% purity) (Figure 2B). Although solubility of the recombinant protein was poor, sufficient soluble protein was obtained to perform an immunoblot-inhibition to further support the designation of the 17 kDa band as oleosin. To this end, serum of a patient (asthma, eczema, and allergy to hazelnut, other tree nuts and peanut) was selected that demostrated significant IgE-reactivity to OAPs (7.1 kU/L) uniquely directed towards the 17 kDa band (serum 9/Figure 3). IgE-binding to natural oleosin and to rCor a 12 was completely inhibited by rCor a 12 (Figure 4). To further support the reactivity to the OAP fraction was caused by oleosin, IgE-responses to rCor a 8 and rCor a 9 were checked by ImmunoCAP and found to be both negative (<0.1 kU/L).

### Biological activity of the OAP fraction in BHR

**Figure 5:**
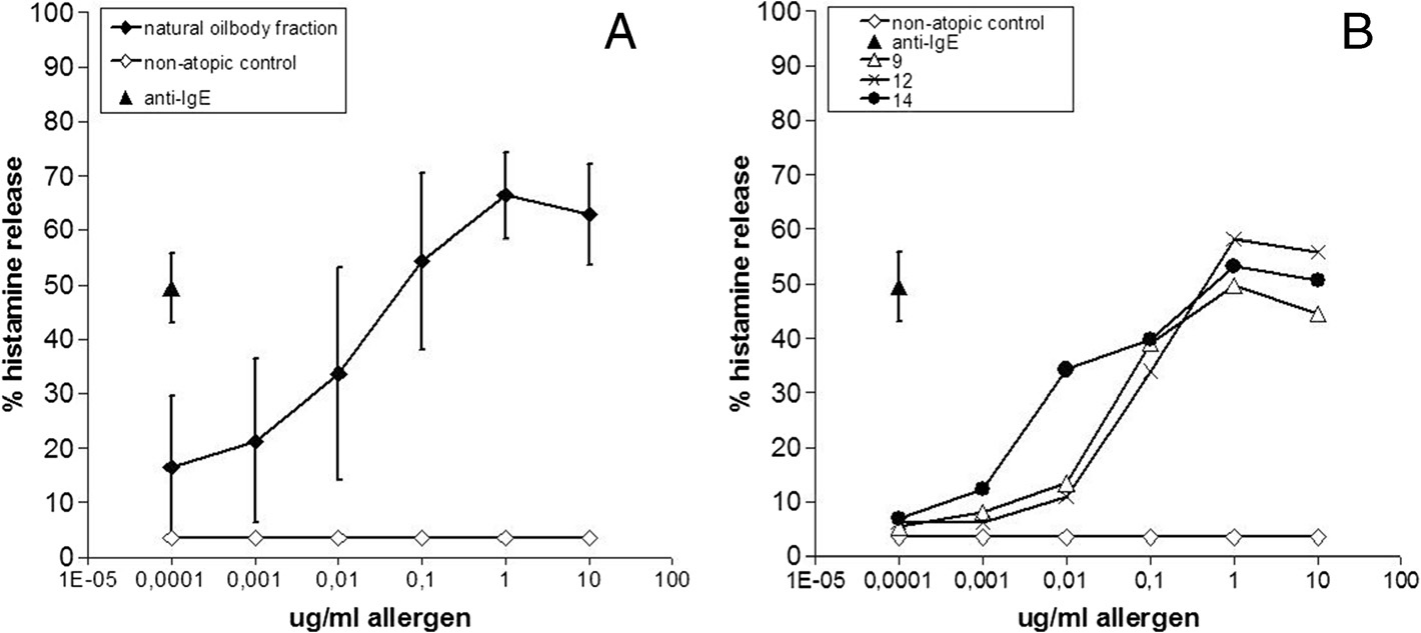
**Basophil histamine release experiment. A**; mean values (+/- standard deviation) of 14 individually tested hazelnut allergic patients to the OAP fraction. **B**; The histamine release from 3/14 individual sera that only/mainly showed reactivity to the Cor a 12 band on blot.

To test the biological activity of IgE antibodies against OAPs, 14 sera were tested by BHR. All showed significant histamine release up to 50-75% with OAPs, starting from a concentration of 100 pg/ml (Figure 5A). Amongst these 14, 3 were uniquely reactive and 2 mainly reactive with the 17 kDa oleosin band, demonstrating that IgE antibodies against oleosin are biologically active (shown for 3 in Figure 5B).

### OAPs in commercial hazelnut SPT reagents

**Figure 6:**
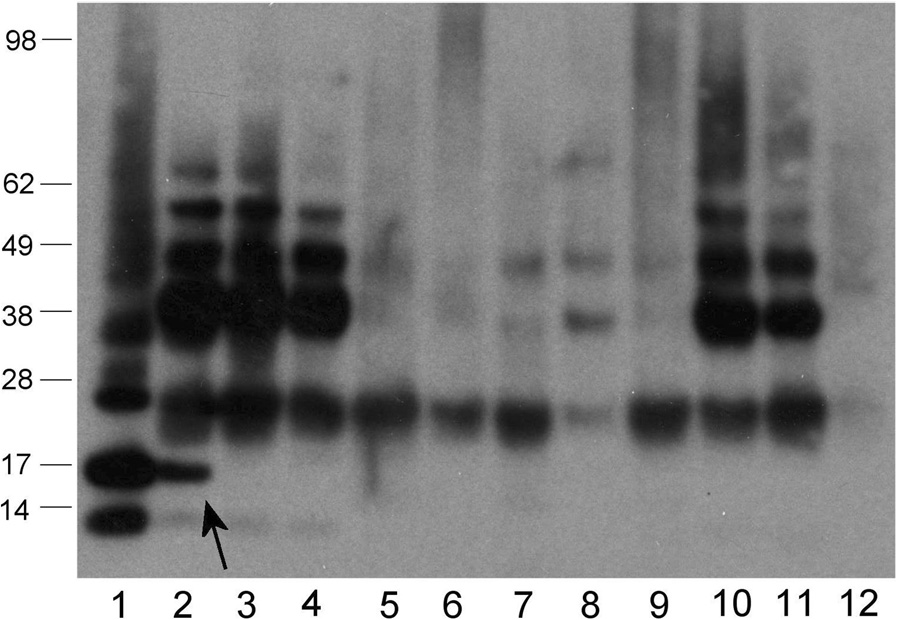
**Immunoblot with rabbit anti-OAP.** 1: natural hazelnut OAP fraction; 2:non-defatted in-house hazelnut extract, 3: defatted in-house hazelnut extract, 4–12: commercially available hazelnut extracts (A-I). The arrow indicates the most IgE-reactive oleosin isoform. Marker sizes are indicated on the left hand side.

**Figure 7:**
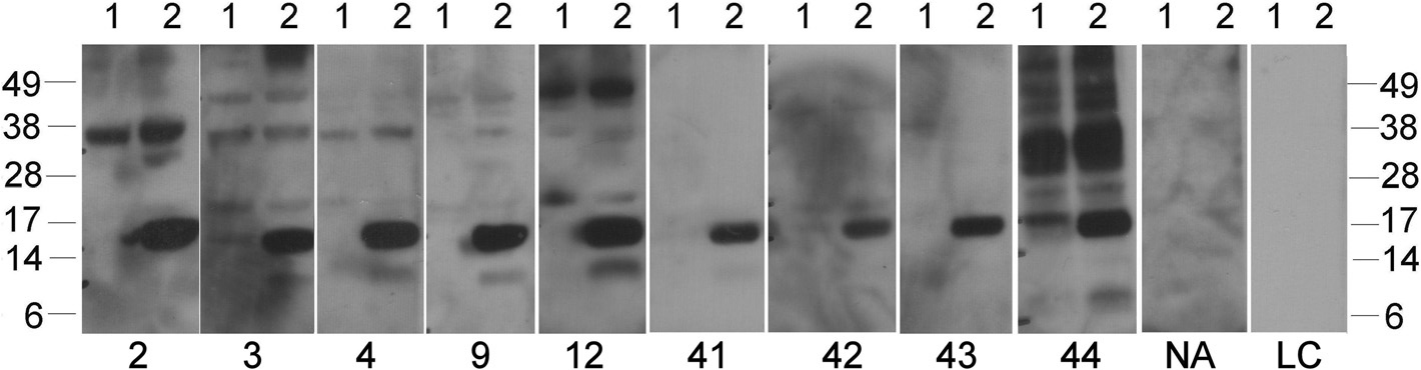
**Immunoblot showing IgE-reactivity of 9 patient sera to a commercially available hazelnut extract (D) unspiked (lanes 1) or spiked (lanes 2) with the hazelnut OAP fraction.** Marker sizes are indicated at both sides; NA: non-atopic control serum; LC: label control.

**Figure 8:**
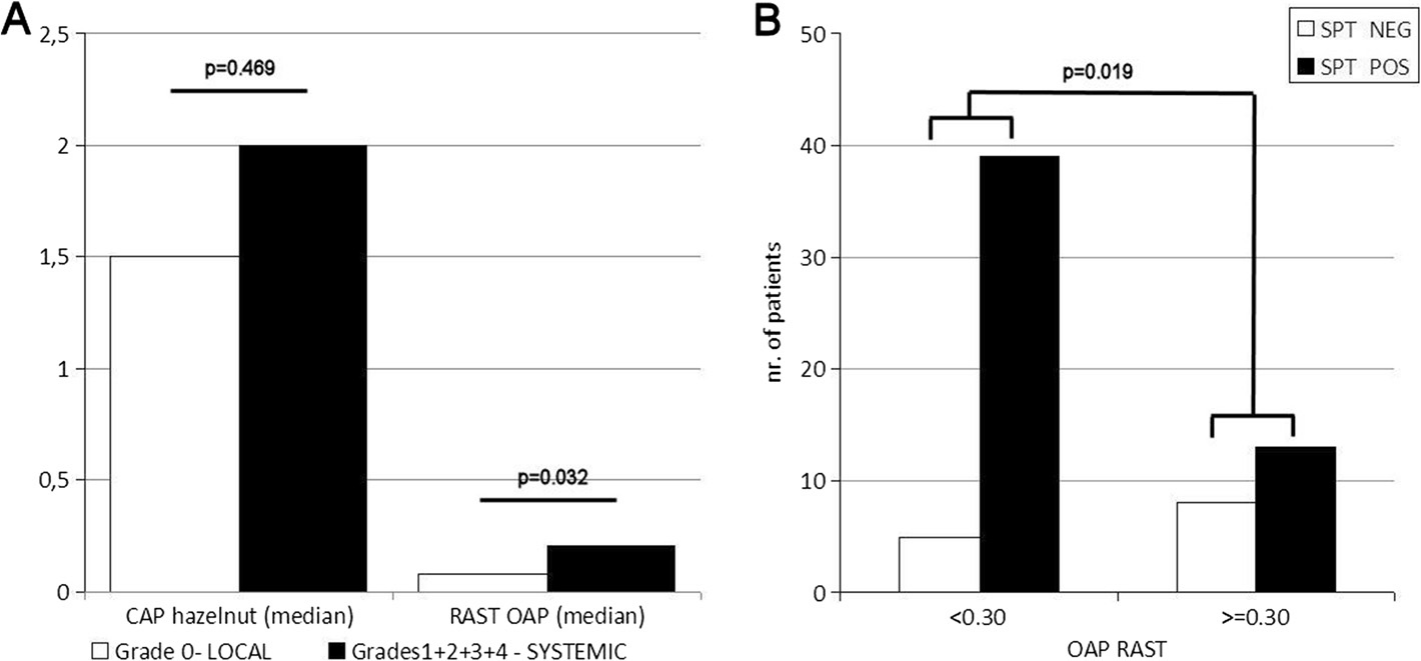
**Correlations between severity grade, hazelnut CAP, OAP RAST and SPT. A**; Correlation between severity grade (grade 0 vs grade 1–4 combined) and hazelnut CAP or OAP RAST. **B**; Correlation between OAP RAST (cut-off 0.3 kU/L) and SPT (pos/neg).

Nine commercially available SPT reagents and an in-house defatted hazelnut extract were evaluated by immunoblot with OAP-specific rabbit antiserum for the presence of OAPs (Figure 6). As positive controls the purified OAP fraction and a non-defatted hazelnut extract were used. Clearly, the 14 and 17 kDa bands were virtually absent in all SPT reagents and the defatted in-house hazelnut extract. The OAP fraction and to a lesser extent the non-defatted extract were positive. Subsequently, we used 9 sera for a spiking experiment of SPT reagent D with OAPs, all had a convincing history of hazelnut allergy. Three of them had a negative/borderline SPT for hazelnut, and for the remaining 6 SPT data to hazelnut were not available. Spiking of the SPT reagent with OAPs convincingly demonstrated that all patients strongly recognized a band slightly under 17 kDa, and some also weakly a band of slightly under 14 kDa, in the spiked but not in the commercial SPT (Figure 7). These bands correspond to the molecular mass of both oleosin bands identified in the OAP fraction. In our group of hazelnut allergic patients from which hazelnut SPT data were available (n = 65/91) a RAST ≥0.30 kU/L to OAPs was significantly associated with a negative SPT outcome (*χ*^2^ 6.3, p = 0.012 (0.019 using Fischer’s exact test), Figure 8B).

### Clinical relevance of sensitization to OAPs

In order to asses whether OAPs may be associated with more severe clinical phenotypes amongst hazelnut allergic patients (n = 91), we performed univariate and multivariate regression analysis, using the clinical characteristics summarized in Table 1. Hazelnut ImmunoCAP values were available for 84/91 subjects with a median value of 2.0 kU/L (IQR 0.5-7.03 kU/L). RAST to the OAP fraction was performed in all of them with a median value of 0.13 IU/ml (IQR 0.07-2.25 IU/ml). The hazelnut CAP and the OAP RAST increased with the severity of the reported reaction (from Grade 0 to 4), and the association was significant for both: OAP RAST (p = 0.014); hazelnut CAP (p = 0.026). When combining grades 1 to 4 (all systemic), the association is only significant for the OAP RAST (p = 0.032) and not for the hazelnut CAP (p = 0.469) (Figure 8A). In the univariate analysis associations with a p <0.10 were found between systemic reactions and OAP RAST (*χ*^2^ 6.8, p = 0.009), pollen allergy (*χ*^2^ 3.8, p = 0.07), and allergic rhinitis (*χ*^2^ 3.0, p = 0.08). However, in the logistic model only the OAP RAST was independently associated with systemic reactions with an OR 4.24 (95% confidence interval 1.34-14.62, p 0.015).

**Table 1:** Descriptive statistics of our patient panel (n = 91)

**Sex (N =90, 1 missing)**	
Male	45 (50%)
Female	45 (50%)
**Severity of reactions (N =78, 13 missing)**	
Grade 0	27 (34.6%)
Grade 1	11 (14.1%)
Grade 2	25 (32.1%)
Grade 3	11 (14.1%)
Grade 4	4 (5.1%)
**SPT response to hazelnut (N = 65, 26 missing)**	
Positive	52 (80%)
Negative	13 (20%)
**Associated atopic diseases**	
Atopic dermatitis	57/81 (70.4%)
Allergic rhinitis	59/81 (72.8%)
Allergic asthma	65/82 (79.3%)
Respiratory allergy (rhinitis and/or asthma)	75/81 (92.6%)
Birch pollen allergy	57/66 (86.4%)
Grass pollen allergy	57/68 (83.8%)
Pollen allergy (grass and/or birch)	64/71 (90.1%)

## Discussion

The first oleosin identified as food allergen was peanut oleosin[26], followed by sesame oleosin[16]. Using a cDNA library screening approach, we reported the preliminary identification of hazelnut oleosins, Cor a 12 and Cor a 13 as allergens [27]. Here we report the isolation of a natural OAP fraction from hazelnut and demonstrate that the 14 and 17 kDa bands therein are oleosins. The 17 kDa band is recognized by IgE antibodies of the majority of a cohort of hazelnut allergic patients. This was supported by mass spectrometric identity confirmation of both 14 and 17 kDa bands and by complete inhibition of IgE binding to the 17 kDa band by rCor a 12. Surprisingly, specific peptides homologous to the reported sequences of both the 14.7 kDa (Cor a 13) and 16.7 kDa (Cor a 12) isoforms of hazelnut oleosin were identified in both 14 kDa and 17 kDa bands. It can therefore not be ruled out that oleosin isoforms exist in long and truncated variants. The detection of oleosin in the 27 kDa band suggests that hazelnut oleosins also exist as dimers. This has been described before by Li and co-workers [39] who showed that identical oleosin molecules can self-associate, both *in vitro* and *in vivo*, to form homo-oligomers.

Oleosins are highly hydrophobic and consequently, once taken out of their oil body environment, poorly soluble proteins. This was observed when isolating OAPs and when purifying rCor a 12 produced in E.coli. Poor solubility hampers accurate evaluation of the immune-reactivity of oleosins. In the present study, we have nevertheless succeeded in establishing the allergenicity of OAPs, and confirming an important role of oleosin as oil body associated major allergen. The hydrophobic nature of OAPs such as oleosins also explains their virtual absence in commercial SPT reagents, them being extensively defatted during their production. The low sensitivity for detection of OAPs may be at the basis of false-negative SPT in patients with a convincing history of hazelnut allergy. It is of great importance to develop soluble recombinant oleosin reagents that can be used for diagnostic purposes, e.g. for spiking current diagnostic hazelnut extracts used for in vitro diagnosis as has been successfully done with rCor a 1 [40]. We are currently working on several strategies to obtain well folded and soluble recombinant reagents, including the production of soluble domains and the use of conjugated highly soluble carrier proteins. Hopefully, these approaches will allow us to firmly establish the role oleosins play in severe allergies to legumes, nuts and seeds.

In this study we have for the first time demonstrated in a group of 91 hazelnut allergic patients that OAPs are significantly and independently associated with systemic reactions to hazelnut, with an OR 4.24 (95% confidence interval 1.34-14.62, p = 0.015). We can not claim with great certainty that (only) oleosins are at the basis of this association, because minor contaminations with Cor a 9 were detected. Indeed some sera showed clear IgE binding at higher molecular mass, potentially pointing towards Cor a 9 recognition. In this study, in most cases we did not have sufficient serum to also analyze IgE responses to other hazelnut allergens such as Cor a 9, an allergen that has been demonstrated to be associated with more severe hazelnut allergy [13]. Another potential limitation of our findings is the retrospective and non-standardized collection of clinical information by different clinical groups, with only 30/91 having DBPCFC-confirmed hazelnut allergy (one of these with mono-sensitization to oleosin on blot). In order to further support the association of oleosin-specific IgE with severe symptoms, purified oleosin has been integrated into the large European multi-center study on component resolved diagnosis of food allergies, EuroPrevall [41]. EuroPrevall has a serum collection of over seven hundred well characterized hazelnut-allergic patients from 12 European countries of which 128 have been challenged by DBPCFC (90 positive) and an additional 22 had anaphylactic reactions. As soon as sufficient purified and/or soluble recombinant allergen is available, the EuroPrevall serum bank [42] will provide us the opportunity to establish the importance of oleosin for hazelnut allergic patients, to study cross-reactivity to other nuts, legumes and seeds, and to further investigate the importance of sensitization to oleosins in regard to the severity of clinical symptoms. In EuroPrevall, all serum samples have already been tested on a battery of hazelnut components including rCor a 9 (11S) and rCor a 14 (2S). Moreover, demographic and clinical data have been collected in a standardized fashion. Together this will allow us to firmly establish whether oleosins are indeed a risk factor for severe hazelnut allergy, independent from Cor a 8 [7], Cor a 9 and/or Cor a 14 [13]. This will guide us on how to further improve allergy diagnostics.

## Abbreviations

BSA: Bovine serum albumin; PBS: Phosphate-buffered saline; PBS-AT: PBS/0.3% BSA/0.1% Tween-20; RAST: Radio-allergosorbent test; SPT: Skin prick test; DBPCFC: Double-blind placebo-controlled food challenge; BHR: Basophil histamine release assay; HRP: Horseradish peroxidase; kU/L: Kilounits of antibody per liter; OAP: Oil body associated protein.

## Competing interests

The authors declare that they have no competing interests.

## Authors’ contributions

LZ-J designed the study, conducted the histamine release experiments, immunoblot experiments, OAP purifications and wrote the manuscript. MF-R analysed and classified the patients according to medical history and performed the statistical analyses. MGTW performed the molecular cloning experiments and produced the recombinant oleosin. JHA isolated the first oleosins and performed molecular cloning experiments. CS, AL, ACK and PS collected all the patient serum, provided detailed medical histories and diagnostic data. PB and GG performed the mass spectrometry analysis and analysed the data, and took part in writing the manuscript. RvR helped to design the study and read and corrected the manuscript. All authors read and approved the final manuscript.
